# The relationship between at-risk foot and all-cause and cardiovascular mortality among the population with diabetes

**DOI:** 10.3389/fmed.2025.1584949

**Published:** 2025-07-21

**Authors:** Zhe Wu, Jin Dong, Meng Li, Chu Chu, Bin Wang

**Affiliations:** ^1^The First Clinical College, Shandong University of Traditional Chinese Medicine, Jinan, China; ^2^Department of Emergency, The Changqing District People's Hospital, Jinan, China; ^3^Department of Endocrine, The Second Affiliated Hospital of Shandong University of Traditional Chinese Medicine, Jinan, China; ^4^Innovative Institute of Chinese Medicine and Pharmacy, Shandong University of Traditional Chinese Medicine, Jinan, China; ^5^Department of Vascular Surgery, The Second Affiliated Hospital of Shandong University of Traditional Chinese Medicine, Jinan, China

**Keywords:** diabetic foot, at-risk foot, peripheral arterial disease, peripheral neuropathy, mortality

## Abstract

**Background:**

Diabetic foot (DF) is a common complication among people with diabetes, typically caused by peripheral neuropathy (PN) and/or peripheral arterial disease (PAD) of the lower limbs. The existing research mainly focuses on cases of diabetic foot ulcers, while the relationship between at-risk foot and all-cause and cardiovascular mortality in the general U.S. population remains unclear.

**Methods:**

This study utilized data from National Health and Nutrition Examination Survey (NHANES) 1999–2004 to conduct a cohort study. At-risk foot are defined as those in diabetic people who have concurrent PAD and/or PN, and without the presence of chronic ulcers in the lower extremities. Kaplan–Meier survival analysis and Multivariable Cox regression models were used to analyze the association between at-risk foot and all-cause and cardiovascular mortality, with subgroup analyses conducted.

**Results:**

A total of 946 participants were included in the study, of which 301 had at-risk foot. The median follow-up time was 190 months. Multivariable Cox regression analysis showed that the all-cause mortality (HR: 2.050, 95% CI: 1.524, 2.758) and cardiovascular mortality (HR: 2.494, 95% CI: 1.809, 3.438) in at-risk foot people were significantly higher than in those without at-risk foot. Additionally, people with ischemic at-risk foot had a higher risk of mortality compared to those with non-ischemic neuropathic at-risk foot.

**Conclusion:**

Patients with at-risk foot in the diabetic population are significantly associated with increased all-cause and cardiovascular mortality. Early diagnosis and intervention of PAD and PN in diabetes people are crucial for reducing mortality risk.

## Introduction

Diabetic foot (DF) is a serious complication of diabetes caused by peripheral neuropathy (PN) and/or varying degrees of vascular disease (1–3). Clinical manifestations primarily include pain, sensory abnormalities, and motor dysfunction in the lower limbs. As the disease progresses, patients often develop chronic, non-healing foot ulcers and may experience gangrene, which can ultimately lead to amputation or even death in severe cases (4, 5). According to the International Diabetes Federation (IDF), over 500 million people globally have diabetes, and by 2045, this number is expected to exceed 700 million (6). As the prevalence of diabetes continues to rise, DF has become a major public health challenge, particularly among aging populations (7). Statistics indicate that approximately 34% of diabetic people will develop DF ulcers (8). About 10% of DF people die within 1 year of ulceration (9, 10), and the five-year mortality rate for DF ulcer people can reach 50–70%, exceeding that of many cancers (4).

We usually call diabetes people with peripheral neuropathy or peripheral arterial disease but without chronic ulcer of lower limbs as “at-risk foot” people. A cohort study of newly diagnosed diabetes patients over 18 years old in the UK showed that the long-term mortality rate of diabetes patients with foot complications was significantly higher (11). However, most of the existing studies focus on people with diabetes foot ulcers in hospitals or clinics (12–15). Research on at-risk foot in the general population is limited, and there is currently a lack of studies examining the relationship between at-risk foot and mortality in the general U.S. population. The National Health and Nutrition Examination Survey (NHANES) is a nationally representative project based on the general population in the United States. All NHANES data is publicly available, allowing global researchers to use appropriate statistical techniques for secondary analysis. Relevant examination data on lower limb diseases are provided in NHANES 1999–2004. Based on this, our study intends to use the lower limb disease related data from NHANES 1999–2004 to explore the relationship between at-risk foot and all-cause and cardiovascular mortality among individuals with diabetes in the general U.S. population.

## Methods

### Data source

**Figure 1:**
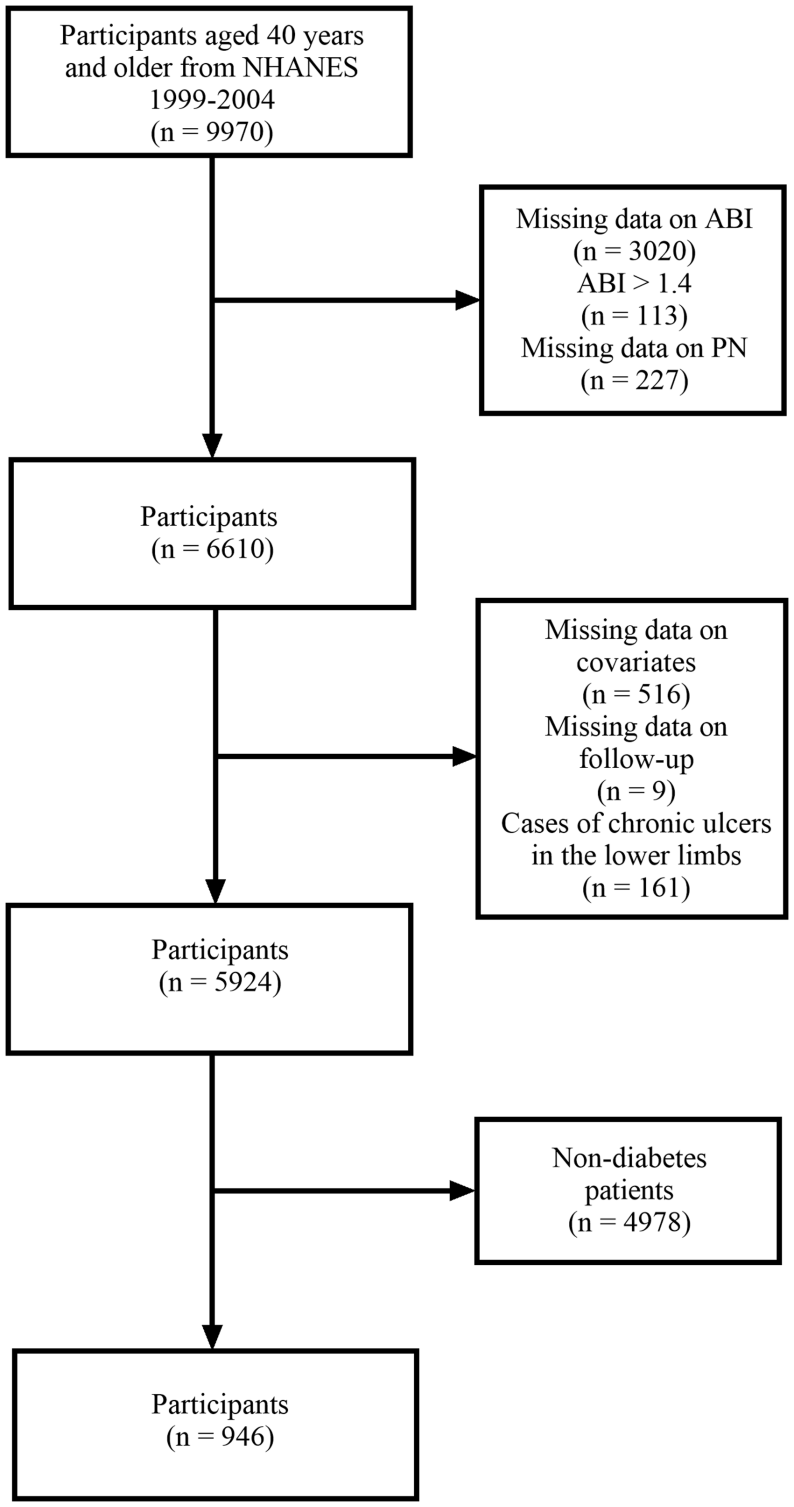
Research flowchart.

NHANES is an ongoing survey conducted by the National Center for Health Statistics (NCHS) to assess the nutrition and health status of the non-institutionalized population in the United States. In NHANES 1999–2004, 9,970 participants aged 40 and older underwent lower limb disease examinations. We initially included all participants who participated in lower limb related disease examinations. Exclusion criteria: (1) Participants without ankle brachial index (ABI) measurement (*n* = 3,020); (2) Participants with ABI > 1.4 (*n* = 113); (3) Participants without available peripheral neuropathy data (=227); (4) Participants without covariate data (*n* = 516); (5) Participants without follow-up data (*n* = 9); (6) Participants with chronic ulcers in the lower limbs (*n* = 161); (7) Participants without diabetes (*n* = 4,978). Ultimately, 946 participants were included in the study (Figure 1).

### At-risk foot

At-risk foot are defined as those in diabetic people who have concurrent peripheral arterial disease (PAD) and/or PN, and without the presence of chronic ulcers in the lower extremities. Diabetes was defined as fasting blood glucose ≥7 mmol/L, random blood glucose ≥11.1 mmol/L, HbA1c ≥ 6.5%, two-hour OGTT blood glucose ≥11.1 mmol/L, or a physician’s diagnosis or use of hypoglycemic medication.

Participants aged 40 and older were required to undergo lower limb disease examinations. Trained health technicians measured the systolic pressure of the posterior tibial artery on both sides as the systolic ankle pressure and the systolic pressure of the right brachial artery as the systolic arm pressure (if the right arm could not be measured or might affect the results, the left arm was measured). The ABI was calculated as the systolic ankle pressure divided by the systolic arm pressure. PAD was defined as an ankle-brachial index <0.9 on either side. In addition, health technicians used a standard monofilament (5.07 Semmes-Weinstein nylon) to test three areas of each foot (the plantar first metatarsal head, the plantar fifth metatarsal head, and the plantar big toe) to check for sensory abnormalities. PN was defined as having at least one insensitive area on both feet. The prevalence of chronic ulcers in the lower extremities was determined through a questionnaire. Participants were asked, “Have you ever had an ulcer or sore on your leg or foot that took more than 4 weeks to heal?” A “yes” response indicated the presence of chronic lower extremity ulcers.

### Outcome assessment

Survival status data and follow-up information for all individuals were collected until December 31, 2019. All-cause mortality was determined using survival data from the National Death Index (NDI). Cardiovascular mortality was identified using ICD-10 codes (I00-I09, I11, I13, I20-I51, I60-I69).

### Covariates

Covariates included age, sex, race, body mass index (BMI), total cholesterol, aspartate aminotransferase (AST), alanine aminotransferase (ALT), smoking history, drinking history, and whether the participant had hypertension, cardiovascular disease (CVD), or chronic kidney disease (CKD). Hypertension was defined as average systolic pressure ≥140 mmHg, average diastolic pressure ≥90 mmHg, or a physician’s diagnosis or use of antihypertensive medication. CVD was obtained through self-reporting. CKD was defined as an estimated glomerular filtration rate <60 mL/min/1.73 m^2^.

### Statistical analysis

Statistical analysis was performed using R Studio (version 4.2.1). All statistical analyses were weighted for population data. Continuous variables were compared using t-tests or Mann–Whitney U tests, and categorical variables were compared using chi-squared tests. Continuous variables that followed a normal distribution are represented as mean (standard error), while those that did not are represented as median (interquartile range). Categorical variables are represented as numbers (weighted percentage). Participants were divided into two groups based on whether they had at-risk foot. Additionally, for practical purposes, at-risk foot people were categorized into two types: non-ischemic neuropathic at-risk foot (Group 1) and ischemic at-risk foot (Group 2) (16). Kaplan–Meier (KM) survival analysis was used to study long-term survival among different groups. A multivariable Cox regression model was employed to estimate the relationship between at-risk foot and all-cause and cardiovascular mortality, with subgroup analyses conducted based on age, sex, race, smoking history, drinking history, and the presence of hypertension, CVD, and CKD.

## Results

### Baseline characteristics

We divided the 946 participants into two groups based on the presence of at-risk foot. There were 301 cases in the at-risk foot group and 645 cases in the non at-risk foot group. Table 1 shows significant differences between the two groups in terms of age, sex, race, ABI, total cholesterol, and the prevalence of hypertension, CKD, and CVD. The median follow-up time for the study was 190 months.

**Table 1:** Population characteristics stratified by at-risk foot.

Variable	Non at-risk foot (*n* = 645)	At-risk foot (*n* = 301)	*p*-value
Age (years)	58.000 (50.000, 67.000)	65.000 (56.000, 73.000)	<0.001
Sex			0.001
Male	333 (51.908)	193 (67.442)	
Female	312 (48.092)	108 (32.558)	
Race			0.030
White	262 (65.713)	131 (70.038)	
Black	129 (11.171)	80 (14.836)	
Mexican American	191 (7.623)	74 (6.062)	
Other Race	63 (15.493)	16 (9.065)	
BMI (kg/m^2^)	30.612 (0.335)	31.244 (0.460)	0.280
ABI	1.103 (0.005)	0.962 (0.016)	<0.001
ALT (μ/L)	29.199 (1.315)	28.548 (2.208)	0.799
AST (μ/L)	26.100 (1.009)	26.582 (1.472)	0.776
Total cholesterol (mg/dL)	212.188 (3.557)	199.553 (3.782)	0.013
Smoking history			0.356
No	292 (45.545)	119 (40.468)	
Yes	353 (54.455)	182 (59.532)	
Drinking history			0.560
No	106 (15.714)	58 (17.581)	
Yes	539 (84.286)	243 (82.419)	
Hypertension			0.016
No	198 (34.861)	71 (23.848)	
Yes	447 (65.139)	230 (76.152)	
CKD			<0.001
No	419 (68.105)	148 (47.117)	
Yes	226 (31.895)	153 (52.883)	
CVD			<0.001
No	519 (82.777)	197 (60.887)	
Yes	126 (17.223)	104 (39.113)	

### KM analysis

**Figure 2:**
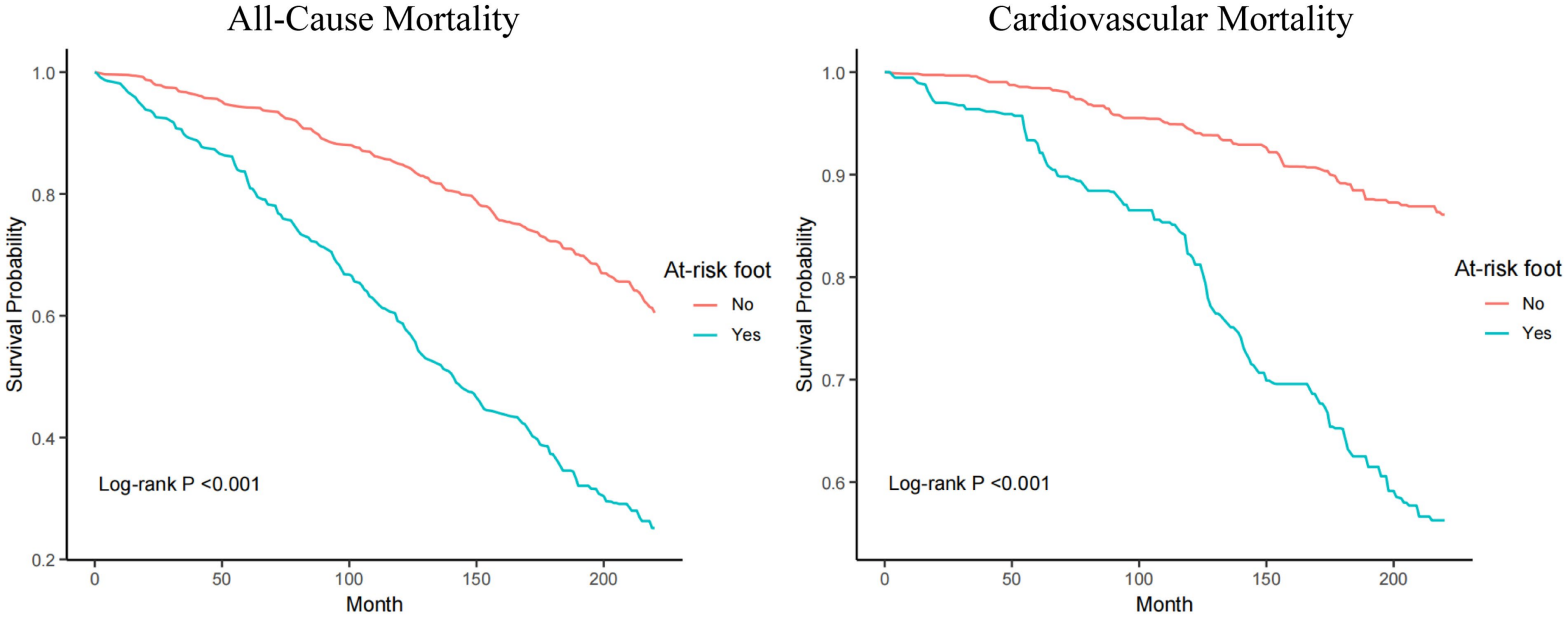
The Kaplan–Meier (K-M) curves of people with at-risk foot.

The Kaplan–Meier (KM) curves demonstrate that both all-cause and cardiovascular mortality rates are significantly higher in at-risk foot people compared to those without at-risk foot (Figure 2).

### Multivariable cox regression

To further explore the relationship between at-risk foot and all-cause and cardiovascular mortality, we conducted multivariable Cox regression. After full adjustment for covariates, a significant positive association remained between at-risk foot and both all-cause mortality (HR: 2.050, 95% CI: 1.524, 2.758) and cardiovascular mortality (HR: 2.494, 95% CI: 1.809, 3.438). Moreover, compared to the non at-risk foot group, the all-cause (HR: 1.732, 95% CI: 1.237, 2.426) and cardiovascular mortality rates (HR: 2.389, 95% CI: 1.654, 3.451) were significantly elevated in the non-ischemic neuropathic at-risk foot group. People with ischemic at-risk foot exhibited even higher all-cause mortality (HR: 2.590, 95% CI: 1.886, 3.557) and cardiovascular mortality (HR: 2.647, 95% CI: 1.653, 4.238) (Table 2).

**Table 2:** Multivariate cox regression analysis of at-risk foot and all-cause and cardiovascular mortality.

Result	Model 1		Model 2		Model 3	
	HR (95%CI)	*p*-value	HR (95%CI)	*p*-value	HR (95%CI)	*p*-value
All-cause mortality	2.798 (2.107, 3.715)	<0.001	2.197 (1.650, 2.924)	<0.001	2.050 (1.524, 2.758)	<0.001
Non at-risk foot (*n* = 645)	Ref	Ref	Ref	Ref	Ref	Ref
Group 1 (*n* = 169)	2.061 (1.504, 2.824)	<0.001	1.793 (1.282, 2.506)	<0.001	1.732 (1.237, 2.426)	0.001
Group 2 (*n* = 132)	4.625 (3.557, 6.015)	<0.001	2.974 (2.256, 3.919)	<0.001	2.590 (1.886, 3.557)	<0.001
Cardiovascular Mortality	3.626 (2.658, 4.948)	<0.001	2.857 (2.119, 3.850)	<0.001	2.494 (1.809, 3.438)	<0.001
Non at-risk foot (*n* = 645)	Ref	Ref	Ref	Ref	Ref	Ref
Group 1 (*n* = 169)	2.867 (1.900, 4.325)	<0.001	2.507 (1.703, 3.691)	<0.001	2.389 (1.654, 3.451)	<0.001
Group 2 (*n* = 132)	5.480 (3.974, 7.555)	<0.001	3.511 (2.404, 5.130)	<0.001	2.647 (1.653, 4.238)	<0.001

Adjusted variables: Model 1: unadjusted. Model 2: age, sex, race. Model 3: age, sex, race, BMI, smoking history, total cholesterol, ALT, AST, hypertension, CKD, CVD.

### Subgroup analysis

**Figure 3:**
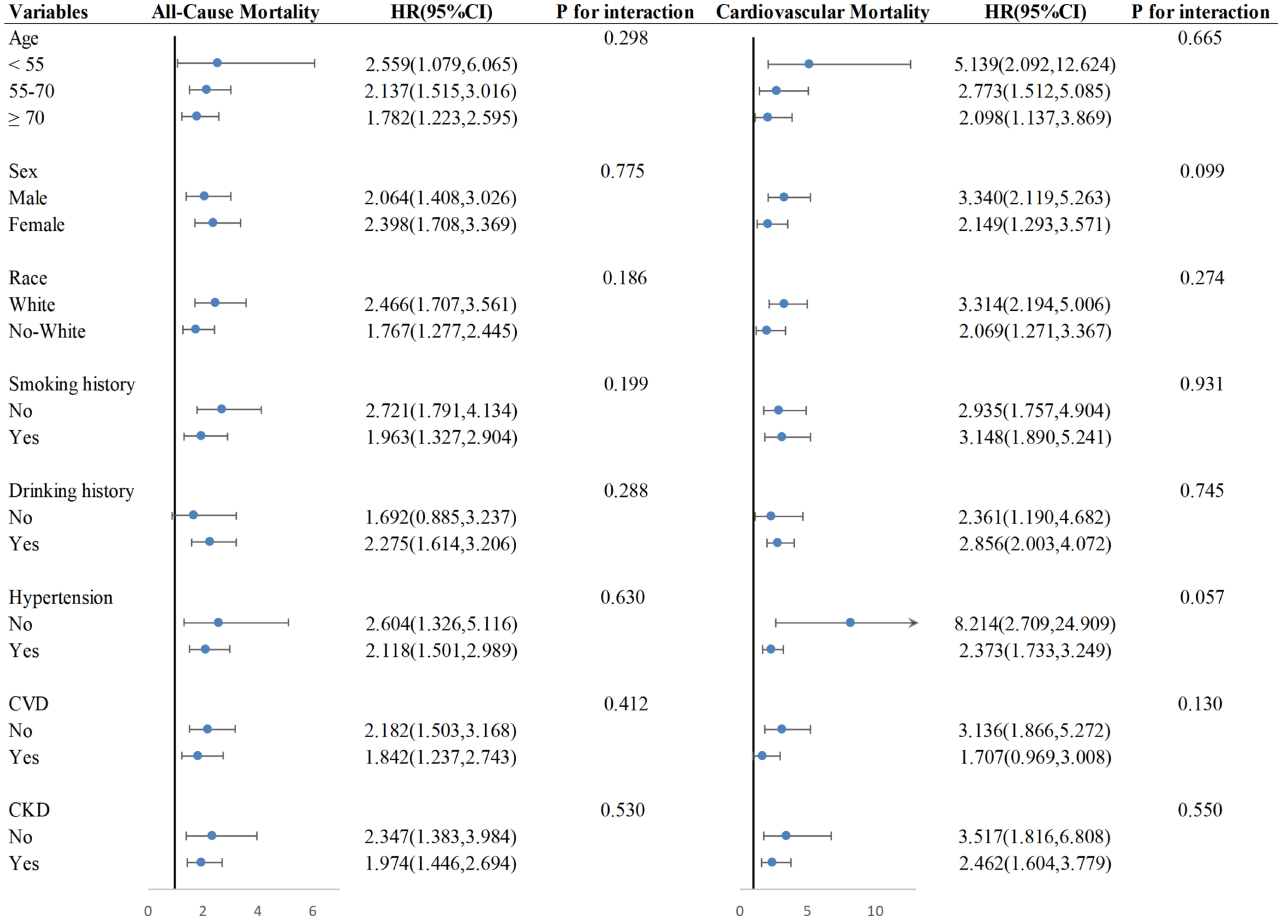
Subgroup analysis.

Adjustments were made for covariates other than grouping variables in subgroup analysis. The results indicate that the positive correlation between at-risk foot and all-cause mortality remains significant in all subgroups except for the subgroup of participants with no drinking history. The relationship between at-risk foot and cardiovascular mortality remained significant in all subgroups except for the subgroup of participants with CVD (Figure 3).

### Sensitivity analysis

After excluding data on deaths occurring within 2 years of follow-up, we re-conducted the multivariable Cox regression, yielding consistent results with the previous findings (Table 3). This indicates that our results are stable.

**Table 3:** Sensitivity analysis.

Result	Model 1		Model 2		Model 3	
	HR (95%CI)	*p*-value	HR (95%CI)	*p*-value	HR (95%CI)	*p*-value
All-cause mortality	2.737 (2.016, 3.717)	<0.001	2.162 (1.588, 2.944)	<0.001	2.026 (1.476, 2.779)	<0.001
Non at-risk foot (*n* = 630)	Ref	Ref	Ref	Ref	Ref	Ref
Group 1 (*n* = 165)	2.125 (1.529, 2.954)	<0.001	1.847 (1.309, 2.605)	<0.001	1.783 (1.261, 2.521)	0.001
Group 2 (*n* = 114)	4.311 (3.234, 5.747)	<0.001	2.798 (2.027, 3.861)	<0.001	2.463 (1.722, 3.524)	<0.001
Cardiovascular Mortality	3.446 (2.473, 4.800)	<0.001	2.780 (2.030, 3.808)	<0.001	2.460 (1.766, 3.428)	<0.001
Non at-risk foot (*n* = 630)	Ref	Ref	Ref	Ref	Ref	Ref
Group 1 (*n* = 165)	2.789 (1.830, 4.250)	<0.001	2.483 (1.687, 3.653)	<0.001	2.389 (1.652, 3.457)	<0.001
Group 2 (*n* = 114)	5.092 (3.578, 7.247)	<0.001	3.359 (2.170, 5.199)	<0.001	2.570 (1.542, 4.282)	<0.001

Adjusted variables: Model 1: unadjusted. Model 2: age, sex, race. Model 3: age, sex, race, BMI, smoking history, total cholesterol, ALT, AST, hypertension, CKD, CVD.

## Discussion

Our study is the first to investigate the association between at-risk foot and all-cause and cardiovascular mortality in diabetic people within the general U.S. population. The results indicate that individuals with at-risk foot among diabetic people have significantly increased risks of all-cause and cardiovascular mortality. Notably, compared to at-risk foot people solely caused by PN, at-risk foot people with PAD face a higher risk of long-term mortality.

Previous studies on DF and mortality have predominantly focused on DF ulcer people, overlooking early DF populations (17–19). Our study indicates that diabetic people with either PAD or PN, even in the absence of chronic lower extremity ulcers, experience significantly increased mortality rates. In fact, DF ulcers do not appear suddenly. Statistics show that the prevalence of PAD among diabetic people is four times that of non-diabetic people (20). Additionally, arterial occlusion in diabetic people often occurs below the knee and is typically characterized by long segment occlusions (21). Moreover, the arteries below the knee are relatively narrow, leading to more severe consequences after occlusion. Insufficient foot perfusion due to ischemia is a significant cause of delayed ulcer healing (22, 23). PN is a primary cause of most DF ulcers, with over 82% of DF people exhibiting this condition (24). Our study also indicates a higher prevalence of PN compared to PAD. PN leads to sensory abnormalities in the foot, causing people to be unaware of excessive or repeated pressure, ultimately resulting in foot ulcers (25–28). When DF people develop ulcers, it usually means that the disease has entered the late stage. Despite undergoing revascularization or neurotrophic therapy, the rates of amputation and ulcer recurrence are still relatively high (29–31). Thus, early screening and diagnosis of DF are crucial.

A key strength of our study is its focus on a general population sample rather than hospital or clinic-based cohorts. Few studies have conducted ABI testing and screening for PN in general populations. NHANES provides an excellent sample for investigating disease risk factors and mortality in a true general population. Most DF people in the general population are in the early stages of the disease and have not progressed to ulceration or gangrene. Studies suggest that targeted treatments for early DF people, such as intensive glycemic control, lifestyle changes, and regular screening for foot complications, can prevent disease progression (32–34). Moreover, we further classified high-risk foot people based on whether they were ischemic, which further improved our results. In addition, Because NHANES is linked with the NDI, our research benefits from a sufficiently long follow-up period and accurate mortality causes with minimal loss to follow-up.

Our findings have several important clinical implications. First, even in the absence of DF ulcers, comprehensive screening for PN and PAD in diabetic people is necessary, with targeted treatment for those with abnormal results to reduce long-term mortality risks. Second, our study highlights the importance of patient education in preventing DF complications. Patients should recognize the significance of regular foot examinations and appropriate foot care, with timely treatment of any foot-related issues to prevent more severe complications, including amputations.

Despite the strengths of our study, several limitations should be considered when interpreting the results. First, our research is observational, limiting the ability to determine the specific causal mechanisms linking at-risk foot to increased all-cause and cardiovascular mortality. Secondly, the sample size related to cardiovascular mortality is relatively small, which may introduce some degree of error in the results.

## Conclusion

In conclusion, our study indicates that at-risk foot is significantly associated with increased risks of all-cause and cardiovascular mortality, particularly among at-risk foot people with PAD. These findings emphasize the importance of early diagnosis and intervention of PAD and PN in diabetes people to reduce the risk of death. Future research should focus on identifying the most effective strategies for managing at-risk foot in the general population to improve patient outcomes and quality of life.

## Data Availability

The raw data supporting the conclusions of this article will be made available by the authors, without undue reservation.
